# The association of appetite and hormones (leptin, ghrelin, and Insulin) with resting metabolic rate in overweight/ obese women: a case–control study

**DOI:** 10.1186/s40795-022-00531-w

**Published:** 2022-04-29

**Authors:** Sara Hajishizari, Hossein Imani, Sanaz Mehranfar, Mir Saeed Yekaninejad, Atieh Mirzababaei, Cain C. T. Clark, Khadijeh Mirzaei

**Affiliations:** 1 Department of Community Nutrition, School of Nutritional Sciences and Dietetics, Tehran University of Medical Sciences (TUMS), Tehran, Iran; 2 Department of Clinical Nutrition, School of Nutritional Sciences and Dietetics, Tehran University of Medical Sciences (TUMS), Tehran, Iran; 3 Department of Epidemiology and Biostatistics, School of Health, Tehran University of Medical Sciences, Tehran, Iran; 4 Centre for Intelligent Healthcare, Coventry University, Coventry, CV1 5FB UK

**Keywords:** Energy expenditure, Obesity, Resting metabolic rate, Appetite, Ghrelin, Leptin, Insulin

## Abstract

**Objective:**

Low resting metabolic rate (RMR), as a risk factor for weight gain and obesity, can be influenced by many factors. Empirical research has confirmed the role of appetite and related hormones in obesity and energy intake. This study aimed to investigate the relationship between appetite and related hormones in overweight or obese Iranian women with normal and hypo RMR.

**Methods:**

This case–control study was conducted on 42 Iranian adult women (21 cases, and 21 controls), aged 18–48 years**.** An impedance body analyzer was used to obtain the body composition and an indirect calorimeter was used to assess the RMR. The Flint questionnaire was used to assess appetite, dietary intake, and physical activity were assessed by FFQ and IPAQ questionnaires respectively, and ELISA kits were used to assess leptin, ghrelin, and insulin hormones.

**Results:**

The results of the study demonstrated a negative association between ghrelin hormone level (β = -0.34, 95%CI = -61.70,-3.86, *P*-value = 0.027) and RMR, and a positive association between insulin hormone level (β = 0.48, 95%CI = 9.38–34.35, *P*-value = 0.001) and RMR. Also, results of the appetite questionnaire showed that, in general, both appetite (β = 0.32, 95%CI = -0.10–2.99 *P*-value = 0.044) and hunger variable (β = 0.30, 95%CI = 0.04–5.87, *P*-value = 0.047) have a positive association with RMR. There was no significant association between leptin levels and RMR.

**Conclusion:**

It is evident that appetite and related hormones have a potential role in promoting a normal RMR.

## Introduction

Obesity is one of the most important health problems in the last century, and whilst its incidence continues to rise [1]. The etiology of obesity is multifactorial and is generally caused by an imbalance between energy intake and energy expenditure [2]. In general, total daily energy expenditure consists of three main components [3], and resting metabolic rate (RMR) accounts for the largest share of total daily energy requirements (approximately 60 to 75%). Numerous factors affect the level of RMR, including age, sex, race, size, body composition, and hormonal status [4–8]. Studies have shown that RMR is an important determinant of meal size and energy intake per day and reflects the physiological need for food intake [9, 10], and is posited as a predictor of fasting hunger, affecting the overall hunger profile [11].

The brain regulates energy homeostasis through neuropeptide signals from adipose tissue and the gastrointestinal tract; for this reason, the gastrointestinal tract is one of the main components of the food and appetite control system [12]. Many intestinal peptides, including ghrelin, are secreted in this pathway [13]. Ghrelin circulates in two forms: Acylated ghrelin (AG) that has orexigenic, obesogenic, diabetogenic features [14, 15], and Unacylated ghrelin (UAG) that has been demonstrated to inhibit these effects [16–19]. Systemic levels of ghrelin in the blood plasma are increased before eating and its secretion is stimulated and enhanced during calorie restriction [20–23]; ghrelin, therefore, plays a role in controlling energy balance, increasing food intake, and reducing energy consumption [24].

The Insulin and Leptin hormones, balance food intake and energy expenditure. Their circulating levels are associated with body fat content [25]. They affect the central nervous system especially the hypothalamus in proportion to their plasma levels where they act to reduce energy intake and stimulate energy expenditure through the leptin receptor [25–27].

Several studies demonstrated the associations between leptin and RMR when adjusting for body composition. Two of these controlled for fat-free mass (FFM), one of them reported a correlation in women but not in men [28], whereas the other found leptin to be correlated to RMR in men only [29]. The third study controlled for fat mass (FM), age, and sex and found an inverse association between leptin and RMR. However, other studies found no significant associations between leptin and RMR [30–33]. Caudwell et al. demonstrated that RMR was a strong determinant of daily hunger, self-selected meal size, and daily energy intake in overweight and obese individuals [9], while Westerterp-Plantenga et al. noted a negative association between RMR and meal frequency in young men [34]. Furthermore, the findings of Johnson, K.O. et al. imply that RMR does not influence appetite [35].

Nonetheless, the relationship between appetite and related hormones with RMR requires further investigation. As studies have yielded conflicting results and did not assess the association in stratified RMR groups. Moreover, they did not evaluate appetite sensation, physical activity, and dietary intakes (micronutrients, macronutrients, and food groups). Thus, we sought to discern whether appetite and hormones differed in overweight/obese women with normal RMR and hypo RMR. To the best of our knowledge, this is the first study of its kind in this field.

## Methods

### Study population, sampling procedures

We conducted a case–control study and enrolled 42 women from Tehran (21 cases and 21 controls), matched for age and BMI, who had been referred to a nutritional laboratory from September 2019 to January 2019. The sample size was determined to recognize the difference of 2 ng (µ_1-_µ_2=_2) between the case and control groups in the Type I error α = 0.05 and the power of 1-β = 0.90. According to a study by Marzullo and colleagues [36], the standard deviation for ghrelin was determined to be 2. Using the above descriptive, the sample size of 21 was calculated, which implied we needed to recruit 21 participants in each group.

**Fig. 1:**
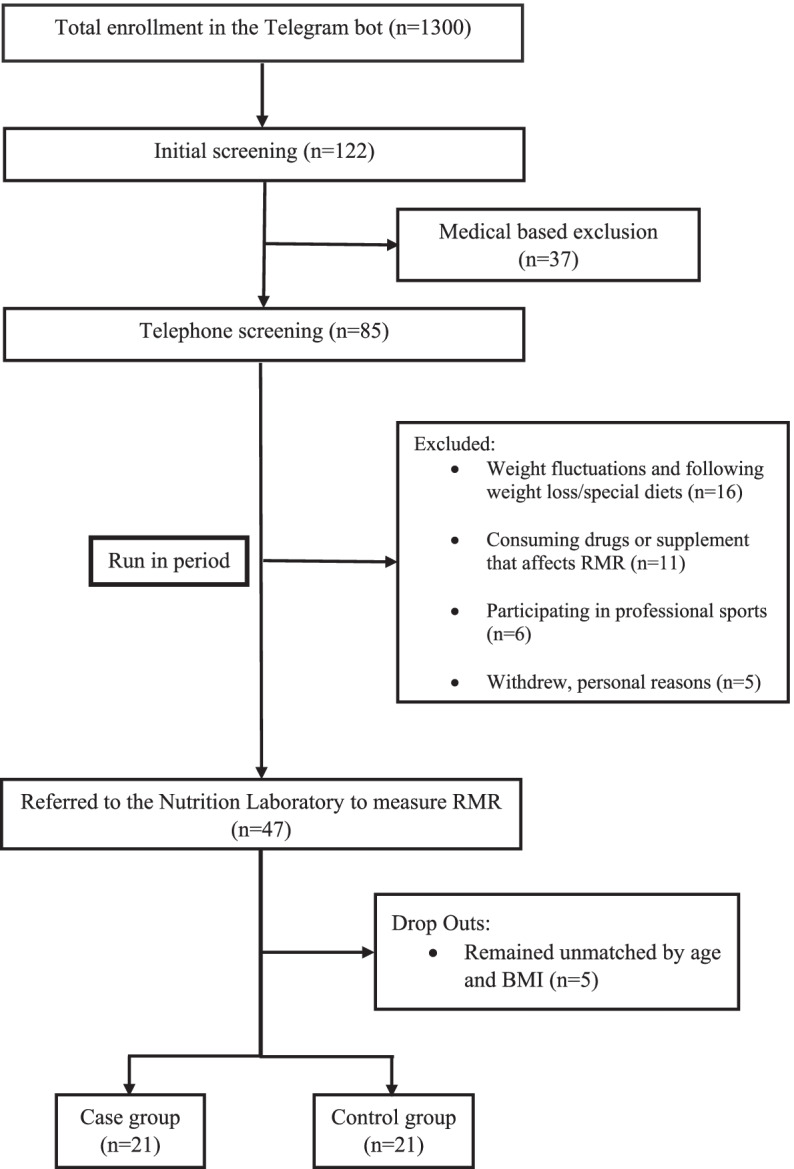
Flow chart diagram of study participants

We designed a Telegram bot, and 1300 volunteers were signed up. Participants were asked to fill out the initial online questionnaire to find out whether they have the basic criteria for inclusion in the study. Out of 1300 volunteers registered, 122 people were eligible samples, and finally, after assessing their medical history, 85 people met the final conditions for study entry (Figure 1). In a monitoring period of 2–3 months, the subjects were followed by phone call to make sure that, they don’t follow special diets and not experiencing weight fluctuations or not participating in any professional sport, to coordinate and control the physical activity we recommended to all participants to keep 20 min of moderate physical activity that is available to everyone and we inquired it by phone call. Also, participants were monitored in terms of consumption of alcohol, drugs, and supplements that affect the rate of metabolism and related hormones. After a follow-up period, 47 individuals were referred to the Nutrition Laboratory of the Faculty of Nutrition and Dietetics located at the School of Health of Tehran University of Medical Sciences for more detailed evaluations. Each person in the case group (low metabolism) was randomly matched with a person in the control group (normal metabolism) with a maximum difference in age ± 2 years and body mass index ± 2 units. Volunteers were randomly selected for the study based on the following inclusion criteria (for both cases and controls): Female gender, 25 ≤ BMI < 40 (obesity and overweight), age over 18 to 50 years, willingness to participate in the study. To ensure comparable data, participants who met any of the following exclusion criteria were excluded: having cardiovascular disease, liver, kidney, thyroid, cancer, diabetes, heart failure, and acute or chronic infections based on patient statements and medical history, consumption of drugs and supplements that affect the rate of metabolism, pregnant, lactating, postmenopausal women and professional athletes, history of weight loss surgeries, history of weight loss diet and weight changes during the last 6 months, drug and alcohol use, following special diets, and reluctance to continue. Participants were told that they could withdraw themselves or their data from the study at any time.

The study protocol was approved by the ethics committee of Tehran University of Medical Sciences (ethics code: (IR.TUMS.MEDICINE.REC.1399.261), and all subjects signed written informed consent prior to participation.

## Measurements

### Resting metabolic rate

In this study, RMR was measured by indirect calorimetry (MetaLyzer 3B-R3 Cortex Biophysik GmbH spirometer, Germany) [37]. Ventilation and gas exchange were calibrated for each individual before each experiment, and the amount of carbon dioxide produced and oxygen consumed per breath was recorded. In this study, a respiratory mask was used, respiratory volume was transferred through a transducer attached to the mask, and gas samples were collected. Resting metabolic rate was assessed in the early morning and after 10 to 12 h of fasting. Participants were asked to refrain from consuming caffeine and exercising vigorously for 24 h before the test. Subjects were tested in the follicular phase of the menstrual cycle, because during the luteal phase of the menstrual cycle, RMR normally rises and is lower during menstruation [38, 39]. The measurements were taken with the patients lying down in a supine position for30 minutes; where the middle 20 min were used for analyses (the first and the last 5 min were ignored). The room was kept at a constant temperature of 22 degrees Celsius, and the validation of this device has been confirmed by several studies [40, 41]. Out of 47 participants who underwent the RMR test, a total of 21 patients were in the case group and 21 patients were in the control group. Three patients with low RMR (case group) and 2 patients with normal RMR (control group) were excluded from the study because they remained unmatched. The criterion for determining low RMR in this study is based on Flancbaum et al. [42], where subjects were defined as “hypometabolic” when their measured RMR was less than 85% of the predicted RMR, based on the Harris and Benedict equation, or “normometabolic” when it was within ± 15% of the predicted RMR. PRMR was also calculated based on the formula of Harris-Benedict [43].

Predicted RMR (Harris-Benedict) = 665 + 9.56 × weight (kg) + 1.84 × height (cm) – 4.67 × age (year).

### Body composition

Body composition, including weight, body mass index (BMI), body fat mass (BFM), fat-free mass (FFM), visceral fat level, visceral fat area, skeletal muscle mass, body fat percentage (PBF%), waist to hip ratio (WHR), waist circumference (WC), and soft lean mass were obtained using a body composition analyzer [44]. To measure the body composition of all participants, in a fasted state, the body analyzer InBody 770 scanner (Inbody Co., Seoul, Korea) was used. Before measurement, according to the instructions of the manufacturer, all participants were asked to remove any metallic objects (e.g., jewelry), and asked to stand barefoot, with minimal clothing, on the metal plate of the device. The operation of the body analyzer was conducted according to the manufacturer's instructions.

### Dietary assessments

A person's usual dietary intake over the past year was assessed by interview, using a semi-quantitative, 147-item food frequency questionnaire (FFQ). Based on this questionnaire, the subjects were asked to report the frequency of their food consumption for each food item on a daily, weekly, monthly, or yearly basis. The reliability and validity of this questionnaire in Iranian populations have already been confirmed [45]. Standard unit sizes and items reported on the household measures were converted to grams using the household measures Guide [46]. The energy content of the food items in the food frequency questionnaire was determined using data from the USDA Food Ingredients Table in the Nutritionist 4 nutrition software database.

### Physical activity

In order to assess the physical activity of the participants, the short form of the International Physical Activity Questionnaire (IPAQ), designed by the World Health Organization, was used [47]. The validity and reliability of this tool have already been evaluated and shown to be acceptable in Iranian adult women. Scores were calculated according to the frequency and time spent on light, moderate, high, and very high-intensity activities, based on a list of common daily activities.

### Appetite

In this study, the Flint appetite questionnaire was used to assess mental feelings about appetite [48]. The validity and reliability of this questionnaire were previously approved [48]. Participants completed the appetite questionnaire in the morning, after the RMR test and body composition analyses (which lasted about 35 min). In this research, four indicators were included; feeling of desire for food, feeling of hunger, feeling of satiety, and feeling of consuming future food. Scoring was conducted in the following manner; participants selected a value from 0 to 100 e.g., I am not hungry at all (zero points), I have not been so hungry so far (100 points). The face validity of the test was checked through questioning the experts of validity [49, 50], and the reliability of the test was ensured through the application of test–retest, reliability (Cronbach Alpha 0.8).

### Blood sampling

From all participants, following 12-h overnight fasting, 10 cc of fasting blood was drawn between 7–10 am. After storage for 30 min at room temperature, a blood clot formed and was centrifuged at 3000 g for 20 min. Serums were stored in clean microtubes in a freezer at -80° C until the analysis was performed.

### Biochemical and hormonal assessments

All hormones were measured by the enzyme-linked immunosorbent assay (ELISA) method. Leptin levels were assessed with an LDN kit (Nordhorn, Germany), with a sensitivity of 0.50 ng/ml, total ghrelin levels were assessed, with a sensitivity of 0.01 ng/ml with Crystal Day Christian Day kit, China, and insulin levels were assessed with an IBT kit (Infinitum biotech, IBT; Netherland), with a sensitivity of 0.11 μU / ml.

Intra- and inter-assay coefficients of variation (CV) reported by the manufacturer were 3.7– 5% and 5.9–5.8% for leptin, CV < 8%, and CV < 10% for total ghrelin, and 3.7–4.2% and 3.7–4.2% for insulin, respectively.

### Statistical analysis

All data were analyzed using SPSS software version 25, where *P*-values less than 0.05 were, *a* priori*,* considered statistically significant. The normality of variable distribution was evaluated using Kolmogorov–Smirnov and Shapiro Wilk tests. Because our data did not show a normal distribution, we used non-parametric tests. The Wilcoxon test was used to determine the difference between quantitative variables between case and control groups. Chi-square test or Fischer exact tests were used to determine the distribution of qualitative variables between case and control groups. Linear regression analysis was used to determine the relationship between appetite and related hormones with RMR, in a crude and adjusted model (adjusted for body fat percentage).

## Results

The present study was conducted on 42 obese and overweight Iranian women, of which, 59.5% were married, 69% occupied, 92.8% had a college education and 52.3% had good economic status.

Baseline participant characteristics are presented in (Table 1). The median (IQR) age, weight, BMI, WHR, WC, BFM, FFM were 34.00 (10) years, 77.25 [16] kg, 29.05(5.6) kg/m^2^, 0.92(0.07), 96.50(14.8) cm, 33.10(12.10) kg, 45.20(7.82) kg. The median age, weight, BMI, WHR, WC, BFM, and FFM were not significantly different among the case and control groups; however, there was a statistically significant difference between the case and control groups in terms of PBF%(*P*-value = 0.027). Also, the median of measured RMR was significantly higher in controls compared to cases, (median: 1541.00, IQR: 232.50 vs median: 1306.00, IQR: 325.0) (*P*-value < 0.001).

Based on the test of qualitative variables (such as physical activity) between the case and control groups, there was no significant difference between the two groups (presented in Table 1), in addition to no significant difference in terms of intake of micronutrients, macronutrients and food groups between the two groups (is presented in Table 2).

In Table 3, the median of studied appetite and hormones are presented. The median (IQR) appetite, leptin, ghrelin, and insulin were 225 [75], 20.65(4.68), 1.97(3.58), and 14.38(6.98). Examination of appetite and leptin, ghrelin, and insulin hormones showed no significant difference between the case and control groups (*P*-value > 0.05).

**Fig. 2:**
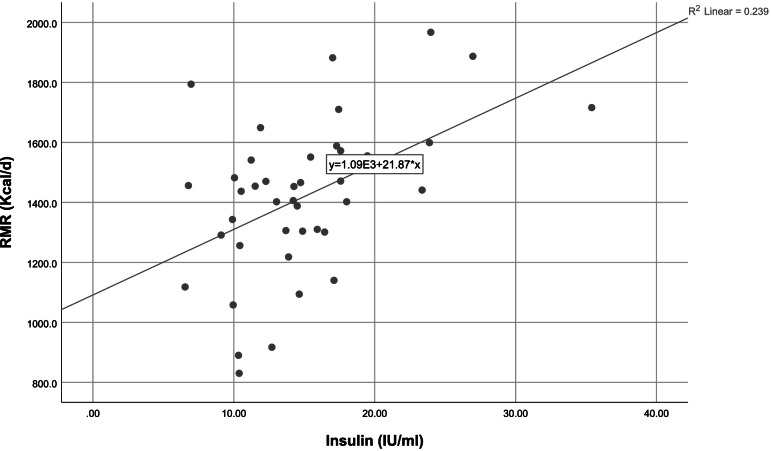
Scatter plot of insulin level and RMR. The line of best fit is indicated. Analysis by simple linear regression showed a significant positive relationship. The *P*-value remained significant after statistical control for PBF %. (*P*-value = 0.001)

**Fig. 3:**
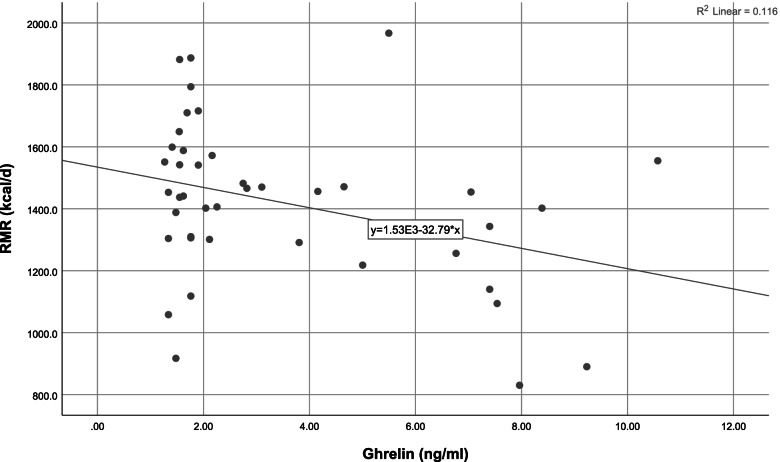
Scatter plot of ghrelin level and RMR. The line of best fit is indicated. Analysis by simple linear regression showed a significant inverse relationship. The *P*-value remained significant after statistical control for PBF %. (*P*-value = 0.02)

To discern an association between appetite and related hormones with RMR, we conducted linear regression, using a crude and adjusted model (presented in Table 4). Our crude result indicated that the insulin hormone levels (β = 0.48 95%CI = 9.38,34.35, *P*-value = 0.001) were positively related to RMR (Fig. 2), and in addition to an inverse association between ghrelin hormone levels (β = -0.34, 95%CI = -61.70,-3.86, *P*-value = 0.027) and RMR (Fig. 3). After adjusting for body fat percentage, the results remained significant, and, in the adjusted model, there was a positive association between appetite (β = 0.32, 95%CI = -0.10,2.99 P-value = 0.04) and RMR. Appetite questionnaire indices and their relationship with RMR are detailed in Table 5. Accordingly, there was a positive significant relationship between the rate of hunger (β = 0.30, 95%CI = 0.09–5.95, *P*-value = 0.04) and RMR, which remained significant after adjusting for the confounding variable. We also investigated the association between RMR and related hormones separately in case and control groups. The results showed that there was a negative relationship between serum ghrelin levels and RMR in the “hypometabolic” group (β = -0.50, 95%CI = -0.01,-0.001, *P*-value = 0.02), after adjusting for PBF%, the results remained significant. While, no association was found between serum ghrelin levels and RMR in the “normometabolic” group (β = -0.12, 95%CI = -0.008,-0.005, *P*-value = 0.59). However the association between Insulin level and RMR was significant in both “hypometabolic” group (β = 0.61, 95%CI = 8.61–36.43, P-value = 0.003) and “normometabolic” group (β = 0.45, 95%CI = 0.80–32.16, *P*-value = 0.04. The results remained significant after controlling for PBF%. Although there was no significant difference between dietary intake and the level of physical activity between the case and control groups, we assessed the association between appetite and related hormones while adjusting for the effects of dietary intake and physical activity, however, the results did not change.

**Table 1:** Characteristics of the subjects in case and control groups

Variables	**Normal RMR group (*n* = 21)**			**Hypo RMR group (*n* = 21)**			*P*-value^*^
	Median,(IQR)	Min	Max	Median,(IQR)	Min	Max	
Age (year)	35.00,(11)	25	45	34.00,(9)	25	47	0.27
**RMR measurement**							
RMR (Kcal/d)	1,541,(232.50)	1291	1967	1306.00,(325.0)	830	1716	** < 0.001**
RQ	0.88,(0.10)	0.83	1.1	0.89,(0.08)	0.79	1	0.68
**Body Composition**							
Height(cm)	163.00,(6)	154	171	159.00,(7)	151	168	0.06
Weight(kg)	80.00,(16)	62.7	102.1	75.40,(13.5)	58.8	105.7	0.28
FFM (kg)	46.8,(6.10)	37.4	57.5	43.10,(9.10)	34.2	56.7	0.06
FM(kg)	34.00,(14.1)	21.4	47.2	33.00,(9.8)	24.2	54.4	0.30
SMM(kg)	25.40,(3.9)	20.2	31.8	23.30,(5.4)	18	31.7	0.06
TBW(L)	34.20,(3.5)	27.4	42.2	32.5,(6.8)	25.1	41.6	0.08
%PBF	41.40,(8.9)	30.6	48.3	44.70,(5.7)	36.1	51.4	**0.02**
WHR	0.93,(0.07)	0.85	1.4	0.92,(0.07)	0.86	1.03	0.49
WC(cm)	98.90,(17.3)	87.1	117.4	93.80,(13.3)	85.5	120.9	0.54
BMI (kg/m^2)^	29.00,(6.9)	26.1	38.1	29.10,(4.7)	25.5	39.8	0.18
**IPAQ**							
High	0(0%)			2(9.5%)			0.21
Moderate	12(57.1%)			14(66.7%)			
Low	9(42.9%)			5(23.8%)			
**Marriage Status**							
Married	14(66.7%)			11(52.4%)			0.53
Single	7(33.3%)			10(47.6%)			
**Education**							
Bachelor	18(85.7%)			18(85.7%)			0.10
Associate Degree	2(9.5%)			1(4.8%)			
Diploma	1(4.8%)			2(9.5%)			
**Occupation**							
Housewife	3(14.3%)			9(42.9%)			0.10
occupied	18(85.7%)			11(52.4%)			
**Economic Status**							
Weak	1(4.8%)			2(9.5%)			0.58
Medium	9(42.9%)			8(38.2%)			
Good	11(52.4%)			11(52.4%)			
**BMI**							
Overweight	11(52.4%)			13(61.9%)			0.75
Obese	10(47.6%)			8(38.1%)			

Quantitative variables were reported with median and interquartile ranges and qualitative variables with number and percentage. *P values resulted from the analysis of the Wilcoxon test for continuous variables and chi-square test for categorical variables. *RMR* resting metabolic rate, *RQ* respiratory quotient, *FFM* fat-free mass, *FM* fat mass, *SMM* Skeletal Muscle Mass, *TBW* Total Body Water, *PBF* Percent of Body Fat, *WHR* waist-to-hip ratio, *WC* waist circumference, *BMI* body mass index, *IPAQ* International Physical Activity Questionnaire

Cut point IPAQ: low < 600 METs, moderate: 600–3000 METs, high > 3000 METs

The economic status is graded based on the number of items listed in the general questionnaire: less than 3 weak, 4–6 medium, and 7–9 good

Body mass index levels are classified according to WHO criteria. Overweight: 25–29.9 (kg / m^2^) Overweight and 30 ≤ Obesity

**Table 2:** Evaluation of food intakes between case and control groups

Variable	Normal RMR group *n* = 21	Hypo RMR group *n* = 21	*P*-value^*^
	Median,(IQR)^**^	Median,(IQR)	
**Food Groups**			
ECereal(g/d)^a^	102.55,(307.30)	351.20,(145.55)	0.09
EFruits(g/d)^b^	321.00,(225.90)	261.40,(314.50)	0.20
EVegetable(g/d)^c^	254.00,(277.50)	336.80,(197.30)	0.21
ELegumes(g/d)	48.30,(47.35)	46.50,(62.90)	0.33
EDairy(g/d)^d^	330.20,(232.85)	320.20,(167.30)	0.49
EWhite meat(g/d)^e^	32.30,(31.10)	50.00,(69.25)	0.18
ERed meat(g/d)s	16.90,(17.50)	21.30,(29.50)	0.20
EFiber(g/d)	37.92,(22.07)	36.76,(16.67)	0.15
**Energy and macronutrients**			
EEnergy(kcal)	2103.24,(630.44)	2279.99,(888.25)	0.18
EProtein(g/d)	81.89,(34.65)	88.00,(30.81)	0.84
ECarbohydrate(g/d)	289.09,(74.74)	327.93,(115.96)	0.14
EFat(g/d)	78.23,(24.92)	76.58,(37.54)	0.63
**Micronutrients**			
EVitamin K (µg /day)	309.13,(352.25)	346.00,(406.21)	0.84
EVitamin D(µg/day)	1.36,(1.77)	1.95,(1.65)	0.20
EVitamin A(RAE/day)	588.41,(393.20)	681.50,(406.90)	0.06
EVitamin E(mg/d)	10.41,(2.79)	9.44(6.05)	0.39
EVitamin C(mg/d)	133.63,(104.88)	119.16,(76.14)	0.61
EFolate(mg/d)	496.77,(162.93)	564.31,(181.71)	0.20
EIron(mg/d)	32.66,(15.07)	33.55,(27.49)	0.41

^**^ Data are presented as median, IQR (interquartile range)

^*^ Resulted from the Wilcoxon test

P-value < 0.05 was considered statistically significant

^a^ Includes whole-grain bread, dietary bread, popcorn, cornflakes, wheat germ, white bread, noodles, pasta, rice, toasted bread, white four, starch, and biscuits

^b^ Includes cherries, apples, raisins or grapes, pears, apricots, tangerines, lemons, oranges, strawberries, mulberries, peaches, nectarines, plums, pomegranates, fresh figs, kiwi, bananas, cantaloupe, watermelon, and dates, melon, greengage, grapefruit, persimmon, cranberry and fruit juice

^c^ Includes carrots, lettuce, cabbage, cauliflower, tomatoes, cucumber, onions, spinach, squash, celery, eggplant, mixed vegetables, green peas, green beans, green pepper, turnip, garlic, and mushrooms

^d^ Includes milk, chocolate milk, cheese, cream, yogurt, cream cheese, and ice cream

^e^ Includes chicken and tuna fish and other fish

**Table 3:** Comparison of the median and interquartile range of the appetite and hormones between case and control

variables	**Normal RMR group (*n* = 21)**			**Hypo RMR group (*n* = 21)**			*P*-value^*^
	Median,(IQR)	Min	Max	Median,(IQR)	Min	Max	
Appetite Questionnaire^**^							
Appetite	250,(88)	125	350	225,(63)	125	300	0.56
**Hormones**							
ELeptin (ng/ml)	21.25,(6.55)	2.21	26.52	20.57,(3.99)	9.69	26.52	0.90
EGhrelin (ng/ml)	1.76,(2.68)	1.27	10.57	2.11,(5.43)	1.34	9.23	0.38
EInsulin (IU/ml)	14.88,(6.21)	6.97	26.96	13.88,(6.42)	6.54	35.4	0.43

^*^P values resulted from the analysis of the Wilcoxon test

^**^ Flint appetite questionnaire was used to assess mental feelings about appetite

Quantitative variables were reported with median and interquartile range (IQR)

**Table 4:** Relationship between appetite and hormones with RMR

Variable		Beta	Standard Error	CI (95%)	*P*-value^**^
^*^Appetite	Crude	0.02	0.76	-0.06,3.01	0.060
	Adjusted	0.32	0.78	-0.10,2.99	**0.044**
**Leptin**	Crude	0.06	8.93	-14.50,21.61	0.693
	Adjusted	0.02	9.65	-18.27,20.79	0.897
**Ghrelin**	Crude	-0.34	14.30	-61.70,-3.86	**0.027**
	Adjusted	-0.34	14.35	-62.66,-4.59	**0.024**
**Insulin**	Crude	0.48	6.17	9.38,34.35	**0.001**
	Adjusted	0.51	6.74	9.55,36.83	**0.001**

Crude Model: In this model, the effect of any of the confounders is not modified

Model 1: In this model, the effect of body fat percentage (PBF %) is adjusted

^**^ Obtained from General Linear Model (GLM)

^*^Flint appetite questionnaire was used to assess mental feelings about appetite

*P*-value < 0.05 was considered statistically significant

**Table 5:** Relationship between variables of appetite questionnaire with RMR

Variables of appetite Questionnaire^*^ (VAS)^**^		**Beta**	**Standard Error**	**CI (95%)**	*P*-Value^***^
**Desire to eat**	Crude	0.25	2.11	-0.81,7.73	0.110
	adjusted	0.24	2.12	-0.88,7.71	0.117
**Hunger**	Crude	0.30	1.44	0.04,5.87	**0.047**
	adjusted	0.31	1.44	0.09,5.95	**0.043**
**Fullness**	Crude	-0.02	1.58	-3.48,2.91	0.859
	adjusted	-0.03	1.59	-3.55,2.89	0.837
**prospective food consumption**	Crude	0.05	1.83	-3.01,4.39	0.710
	adjusted	0.03	1.89	-3.42,4.23	0.832

^*^Flint appetite questionnaire was used to assess mental feelings about appetite

^**^Visual analogue scale VAS, 100 mm in length with words anchored at each end, expressing the most positive and the most negative rating, were used to assess Desire to eat, hunger, fullness, and prospective food consumption

Scoring was conducted in the following manner; participants selected a value from 0 to 100 e.g., I am not hungry at all (zero points), I have not been so hungry so far (100 points)

Model 1: In this model, the effect of body fat percentage (PBF %) is adjusted

^***^ Obtained from General Linear Model (GLM)

*P*-value < 0.05 was considered statistically significant

## Discussion

Our novel approach, including assessing the relationship between stratified RMR groups matching by BMI and age, with both appetite sensation and related hormone levels in a well-characterized case–control study in overweight/obese healthy women., produced data suggesting that there was no significant difference between the median appetite and hormones of leptin, ghrelin, and insulin between the two groups with normal metabolism and impaired metabolism. However, there was a significant positive association between appetite and the rate of hunger with RMR. And also, a positive association between insulin levels with RMR, and a negative association between ghrelin levels with RMR, was found.

We also measured physical activity in the participants. Based on the systematic review and meta-analysis by MacKenzie-Shalders et al., resistance exercise significantly increased RMR when compared to a control group as measured by indirect calorimetry [51]. Physical activity can increase energy expenditure severalfold depending on the volume, mode, and intensity [52]. In our study, however, there was no significant difference in the level of physical activity among the case and control groups, as we coordinate and control it. We also assessed dietary intakes in the study population. Based on previous investigations, adherence to diets lower in carbohydrates and higher in fat and protein were linked with higher RMR [53, 54]. In addition, there was no significant difference in the dietary intakes between the two groups.

The results of the appetite questionnaire indicated a significant direct relationship with RMR. Also, in examining the indicators of the appetite questionnaire [48] with RMR, we found that there was a positive relationship between hunger and RMR, which remained significant after adjusting for the confounding variable, which was concordant with Caudwell et al. [9]. However, Johnson, K.O. et al. findings imply that appetite is linked to FFM rather than RMR [35]. These discrepancies might be due to variations in methodology and design. The obesity paradox highlights that, in people with obesity, despite the higher FM and energy stored in the body (relative to normal-weight individuals), the hunger and the tendency to eat are high [55]; interestingly, it has been posited that, due to the high FM, lean mass (FFM) is also high and FFM is one of the important determinants of RMR [8], and consequently, the higher RMR level corresponds to a higher rate of hunger and appetite [9, 10, 56, 57].

In the present study, the hypothesis that the serum level of leptin hormone is associated with RMR in the study population was not confirmed. There is evidence to suggest that leptin is involved in regulating energy metabolism mainly through its effects on the cardiovascular system and brown adipose tissue thermogenesis via the hypothalamus [58].

However, in our study, there was no difference in leptin between the two groups, and no significant association was observed between this hormone and RMR, which was correspondent with the results of the study by Neuhauser et al.[59]. Human studies examining the relationship between leptin hormone and RMR have yielded conflicting results, some of which have reported no association between RMR and leptin levels in adults [60–62], some studies have reported a positive association [28, 29], and some a negative association [63]. One of the reasons for these contradictory results may be attributed to differences in procedure. In studying the relationship between leptin and RMR, body composition, FM, and FFM are important variables that must be considered [64]. While many of the aforementioned studies reported a positive association, they did not consider these variables. In fact, one of the most important determinants of serum leptin is body fat [62, 65] and also, serum leptin concentration is closely associated with BMI [66], thus, in our study, this may explain why no a difference in leptin levels was seen between the two groups.

We found a negative relationship between serum ghrelin levels and RMR in the whole population study. There is empirical evidence to suggest that ghrelin is involved in energy metabolism, especially in regulating food intake [15, 22, 67–74].

Our results indicated a significant inverse relationship of the ghrelin hormone with RMR in the study population, which is congruent with the results of Pierre et al. [75]. Indeed, Pierre et al., investigated the relationship between RMR and ghrelin levels in normal-weight samples and implied that ghrelin may serve as a biomarker of increased energy efficiency (*i.e.* lower energy expenditure) in humans. The relationship between ghrelin and energy metabolism levels in obese and overweight people evidently needs further investigation [76].

Our analysis conducted separately in stratified RMR groups showed that there is a negative relationship between serum ghrelin levels and RMR only in the “hypometabolic” group. And no association was found between serum ghrelin levels and RMR in the “normometabolic” group. However, Marzullo et al. have shown different results from our research, they suggested that ghrelin secretion is decreased in obesity in cases of impaired energy expenditure [36]. Discrepancies in results might be due to variation in the criterion for determining low RMR and normal RMR. And also Marzullo et al. reported this unexpected association in terms of active ghrelin (Acylated Ghrelin) not total ghrelin level.

One might hypothesize that ghrelin is expected to intervene endogenously to improve impaired energy metabolism. These hypotheses raise the possibility that the metabolic effects of ghrelin may extend beyond the regulation of satiety, food intake, and substrate oxidation, and may play a role to improve energy efficiency in overweight and obese women.

The present study demonstrates that the serum level of insulin hormone is positively associated with RMR in two groups with normal and impaired metabolism, which is concordant with Drabsch et al. and Huang et al. [77, 78]. Homeostatic regulation of glucose is managed through the action of the insulin hormone in adipose tissue, muscle, and liver tissue [79]. In general, one of the main functions of insulin is to focus on tissues that regulate energy metabolism [80].

In fact, the results showed that, the higher the level of insulin hormone, the higher the RMR, which is consistent with Ravussin et al., through the Euglycemic Insulin clamp technique the thermic effect of insulin and glucose injection can be studied in detail [81]. The physiological mechanisms responsible for the relationship between RMR and Insulin level are poorly known. Nevertheless, several proposed explanations include: elevated protein turnover, activated substrate cycle, increased gluconeogenesis, and sympathetic nervous system activity, all potentially caused by hyperinsulinemia [82].

Considering the strengths of the current study, to our knowledge, this study evaluated the relationship between appetite and related hormones with RMR in obese/overweight women by matching two important confounders, age, and BMI for the first time. Moreover, RMR was measured by indirect calorimetry, which is considered as the gold standard for measuring energy expenditure [3]. Also, all participants were examined when they were in the follicular phase, thus reducing the likelihood of the menstrual cycle impacting our results [38, 39]. In addition, we measured each participant's appetite, body composition, and physical activity using established and validated methods. Several limitations in our study should be noted. In the present study, no causal inferences could be drawn owing to the case–control design. Furthermore, the study only looked at overweight/obese women, it would be better to also have a normal weight control group to compare the RMR result, and while we matched cases and controls by age and BMI, there could be some other factors that skewed or confounded our results. Additionally, we used FFQ to record dietary intakes, and reported values from FFQ are subject to substantial errors. However we tried to minimize these problems with the help of experts, and questionnaires were completed in the presence of a trained nutritionist. Although the sample size was similar to that of earlier studies [36], and we calculated the needed sample size to detect the relationship between RMR and related hormones, it would be better to have a larger sample size**.**

## Conclusion

In conclusion, the results of this study showed that there is a significant positive relationship between insulin levels and appetite sensation with RMR in the study population. No association was found between leptin levels and RMR. And a negative association between ghrelin levels with RMR in the “hypometabolic” group. Although speculative, it seems that serum ghrelin levels may play a role in the regulation of energy homeostasis by intervening endogenously to improve the state of impaired energy balance and increase energy efficiency.

## Data Availability

The authors confirm that the data supporting the findings of this study are available within the manuscript and in the included tables.

## Abbreviations

AG: Acylated ghrelin
BFM: Body Fat Mass
BIA: Bioelectrical Impedance Analysis
BMI: Body Mass Index
CV: Coefficients of variation
EE: Energy Expenditure
FFQ: Food frequency questionnaire
FFM: Fat-Free Mass
FM: Fat Mass
IPAQ: International Physical Activity Questionnaires
RMR: Resting Metabolic Rate
REE: Resting Energy Expenditure
UAG: Unacylated ghrelin
